# Cell Wall Pectin and its Methyl-esterification in Transition Zone Determine Al Resistance in Cultivars of Pea (*Pisum sativum*)

**DOI:** 10.3389/fpls.2016.00039

**Published:** 2016-02-01

**Authors:** Xuewen Li, Yalin Li, Mei Qu, Hongdong Xiao, Yingming Feng, Jiayou Liu, Lishu Wu, Min Yu

**Affiliations:** ^1^College of Resources and Environment, Huazhong Agricultural UniversityWuhan, China; ^2^Department of Horticulture, Foshan UniversityFoshan, China

**Keywords:** pea, aluminum sensitivity, transition zone, cell wall pectin, degree of pectin methyl-esterification, pectin methylesterase activity

## Abstract

The initial response of plants to aluminum (Al) is the inhibition of root elongation, while the transition zone is the most Al sensitive zone in the root apex, which may sense the presence of Al and regulate the responses of root to Al toxicity. In the present study, the effect of Al treatment (30 μM, 24 h) on root growth, Al accumulation, and properties of cell wall of two pea (*Pisum sativum* L.) cultivars, cv Onward (Al-resistant) and cv Sima (Al-sensitive), were studied to disclose whether the response of root transition zone to Al toxicity determines Al resistance in pea cultivars. The lower relative root elongation (RRE) and higher Al content were founded in cv Sima compared with cv Onward, which were related to Al-induced the increase of pectin in root segments of both cultivars. The increase of pectin is more prominent in Al-sensitive cultivar than in Al-resistant cultivar. Aluminum toxicity also induced the increase of pectin methylesterases (PME), which is 2.2 times in root transition zone in Al-sensitive cv Sima to that of Al resistant cv Onward, thus led to higher demethylesterified pectin content in root transition zone of Al-sensitive cv Sima. The higher demethylesterified pectin content in root transition zone resulted in more Al accumulation in the cell wall and cytosol in Al-sensitive cv Sima. Our results provide evidence that the increase of pectin content and PME activity under Al toxicity cooperates to determine Al sensitivity in root transition zone that confers Al resistance in cultivars of pea (*Pisum sativum*).

## Introduction

It has been estimated that approximately 50% of the potentially arable lands of the world are acidic soils (Kochian, 1995), where crop productivity is limited by a range of growth-limiting factors related to soil acidity. Aluminum (Al) toxicity is a major limiting factor for plant growth and development in acid soils. The first symptom of Al toxicity is the inhibition of root elongation, which can be measured within hours or less after the roots are exposed to excess Al supply (Llugany et al., 1995; Blamey et al., 2004, 2005). The inhibition of root elongation is usually used for screening Al resistance in plant species and cultivars, which is positively correlated with Al accumulation. The amount of Al accumulation in roots is determined by both the binding sites of plant cells as well as the capacity of rhizo-detoxification by the exudation of organic acids (Jorge and Arruda, 1997; Stass et al., 2008). The release of organic acids induced by Al stress confers to Al-resistance of cultivars in quite a few of plant species, such as maize (Jorge and Arruda, 1997), common bean (Shen et al., 2002), wheat (Stass et al., 2008) and rice (Shi et al., 2007). However, pea (*Pisum sativum* L.) cultivars are not included. There is little difference in the release of organic acids in cultivars with different Al sensitivity (Kobayashi et al., 2004). We guess that Al sensitivity of pea might relate to the other mechanisms, such as the action of Al adsorption on the cell wall.

When the roots are exposed to Al, cell wall is the first target of Al accumulation (Blamey et al., 1993; Kobayashi et al., 2004; Sivaguru et al., 2006; Horst et al., 2007). Studies have shown that about 85–90% of Al is accumulated in the cell wall of barley root apices (Clarkson, 1967), and about 99.9% in *Chara coralline* (Rengel and Reid, 1997). Pectin, a major component of cell wall, has large numbers of negatively charged carboxylic groups which are considered to be the primarily binding sites of Al (Blamey et al., 1990; Chang et al., 1999; Taylor et al., 2000; Wang et al., 2004), even though evidences have been found recently that hemicellulose is an alternative binding site of Al in rice (Yang et al., 2011a). Differences of Al resistance are negatively related to the increased content of pectin in cultivars of rice (Yang et al., 2008) and maize (Eticha et al., 2005a). It is very interesting to know whether it can be applied for cultivars of pea (*Pisum sativum* L.) (Kobayashi et al., 2004) and common beans (*Phaseolus vulgaris* L.) (Rangel et al., 2007), which has a relatively higher content of pectin in the primary cell wall and is very sensitive to Al toxicity in comparison to rice and maize. Actually, majority of the binding sites of pectin is contributed by the action of pectin methylesterases (PME). It is widely accepted that pectin is synthesized in the Golgi and then secreted into the wall as highly methylesterified forms. The highly methylesterified pectin is demethylesterificated by PME with the release of carboxyl groups (Micheli, 2001). Carboxyl groups in the pectin is generally considered to be the main sites for binding Al, and thus its content determines Al sensitivity / resistance in several plant species, e.g., rice (Yang et al., 2008), maize (Eticha et al., 2005a), *Solanum tuberosum* L. (Schmohl et al., 2000). It is intriguing how pectin and PME cooperate to determine Al sensitivity in cultivars of pea with different Al resistance.

Root can be longitudinally divided into zones with different structure and function: root cap, meristem, transition zone, elongation zone, and mature zone (Baluška et al., 1996; Verbelen et al., 2006). Root transition zone is defined recently the root zone between meristem and elongation zone (Baluška et al., 1996, 2001; Verbelen et al., 2006). A number of data suggest that the transition zone is some kind of sensory zone, enabling the growing of root apex (Baluška et al., 1994, 1996). Several studies have shown that the transition zone is the most Al-sensitive zone in the root apex (Sivaguru and Horst, 1998; Kollmeier et al., 2000; Illéš et al., 2006). Baluška et al. (1996) comments that the cells in the transition zone are in a critical preparatory phase based on the synthesis of materials for new tonoplast and plasma membranes, cell wall components, new enzymatic complexes, and cytoplasmic structures. Cell wall pectin, the primary target of Al, is processed by PME after its production and release to apoplast, it is thus hypothesized that cell wall pectin and PME may be responsible for Al sensitivity in root transition zone and Al resistance in cultivars of pea.

In our previous studies about the root zones of pea, 0–1 mm is the cap and meristematic zone, 1.0–2.5 mm is the transition zone, 2.5–5.0 mm is the elongation zone and 5.0–10.0 mm is the maturation (Supplementary Table S1). A detailed research focusing on pectin and Al accumulation is compared in the four root segments in Al-sensitive and Al-resistant cultivars of pea. The objective is to disclose the significance of pectin content and its degree of methyl esterification in determining Al resistance in different cultivars.

## Materials and Methods

### Plant Materials and Growth Conditions

The procedure for pea germination was modified according to Yu et al. (2006). Seeds were immersed in 5.25% sodium hypochlorite for 30 min, and rinsed six times with de-ionized water. Seeds were soaked in 2 mM CaCl_2_ solutions for 8 h and then evenly spread on the mesh screen of the mist culture device with 60 s mist produced every 8 min for 48 h at 24°C. Uniform seedlings, with root lengths ranging from 2 to 3 cm, were selected and transferred to 1/4^th^ Hoagland solution for 4 days under growth chamber at a 16 h (26°C) / 8 h (24°C) day/night regime. Then the seedlings were treated with 30 μM AlCl_3_ (containing 0.5 mM CaCl_2_, 25 μM H_3_BO_3_, pH 4.5) solution for 24 h after pre-adaptation in pH 4.5 (containing 0.5 mM CaCl_2_, 25 μM H_3_BO_3_) circumstance for 8 h. The lateral roots at about 1–2 cm length were used in the study. The lateral roots were neatly placed on a plastic plate with scale, and segments were obtained by hand with sharp razor blades.

### Effect of Al on Root Growth

The entire roots (20 plants for each treatment) were scanned using a root scanner (Epson Expression 11000XL) after rinsing in deionized water. Lateral root length was analyzed with WinRHIZO Pro software before and after Al treatment. The relative root elongation (RRE) was calculated using the following formula: the root elongation under Al treatment/the root elongation in Al-free control × 100.

### Cell Wall Preparation

Cell wall materials were extracted according to the procedure of Heim et al. (1991) and Hoson et al. (2003) with minor modifications. Roots (100 root segments for one replicate) were collected and homogenized, then the homogenates were centrifuged at 15,000 × *g* for 10 min. The precipitate was washed three times with 10 volumes of 80% ethanol and once with 10 volumes of methanol: chloroform mixture (1:1 [v/v]), followed by 10 volumes of acetone. The supernatant of each extracts was discarded and the final pellet freeze-dried. The dried powder was considered as crude cell wall and stored at 4°C for further use.

### Measurement of Al Content

Content of Al in roots (0–10.0 mm, 30 root tips for one replicate), root segments (0–1.0, 1.0–2.5, 2.5–5.0, 5.0–10.0 mm, 50 root segments for one replicate) and cell wall (100 root segments for one replicate) was extracted by 2 M HCl for 48 h with occasional shaking. Content of Al in the extracts was determined by inductively coupled plasma-atomic emission spectrometry (ICP-AES, IRIS-Advantage, Thermo Elemental, Waltham, MA, USA).

### Pectin Determination

Pectin was extracted in crude cell wall powder by 50 mM Na_2_CO_3_ containing 20 mM CDTA (1,2-Diaminocyclohexane-N,N,N′,N′-tetraacetic acid monohydrate). The extracts were centrifuged (15,000 × *g*, 15 min) and the supernatant was the pectin extracts. Galacturonic acid (GalA) content in each pectin extracts was assayed according to the method of Blumenkrantz and Asboe-Hansen (1973). GalA was used as a calibration standard. thus, the pectin content was expressed as GalA equivalents.

The degree of pectin methyl-esterification was measured by colorimetric method (Louvet et al., 2011). Hundred micro liter pectin extracts were saponified by 50 μL 1.5 M NaOH solution for 30 min and surplus alkaline was neutralized by 55 μL 0.75 M H_2_SO_4_. The methanol produced from saponification reaction was determined by colorimetric method, modified from Anthon and Barrett (2004) and Yang et al. (2008). The degree of pectin methyl-esterification was calculated as moles of methanol produced from per mol of galacturonic acid.

### PME Activity Assay

For extraction of PME, different root segments (50 root segments for one replicate) were homogenized in ice-bath and suspended in an extraction buffer containing 100 mM Tris and 1 M NaCl (pH 7.5, pre-cold at 4°C), the suspension was vortexed repeated (20 s for 20 min each) for 1 h. Extracts were centrifuged (15,000 × *g*, 10 min, 4°C) and PME activity was determined in the supernatant following the method of Anthon and Barrett (2004) with minor modification. The incubation contained: 100 μL of 100 mM Tris-HCl (pH 7.5), 0.4 mg/mL of pectin or 100 μL of 100 mM Tris-HCl (pH 7.5) as blank, 100 μL enzyme crude, 40 μL of MBTH (3 mg/mL), 10 μL of alcohol oxidase (AO, 0.01 units/μL). After the addition of AO, the samples were incubated for 20 min at 30°C and then 200 μL of a solution containing 5 mg/mL of ferric ammonium sulfate and sulfamic acid were added to terminate the reaction. After 20 min at room temperature, 550 μL of water was added and A_620_ determined.

### Morin Staining

Roots were stained in 0.01% morin for 30 min (Zhu et al., 2013) after Al treatment, and then rinsed with de-ionized water. Free-hand sections were made with sharp razor blades. The whole root tips and the cross-sections of the different regions were examined and photographed immediately. The green fluorescence signal was observed respectively using an Olympus IX71 fluorescence microscope and a Laser-Scanning Confocal Microscope (LSCM, FV1000, Olympus). At least 5 roots and 10 sections were images for each treatments, and fluorescence intensity was measured with the open source software Image-J.

### Statistics

Random sampling was arranged and each experiment was repeated at least three times. Duncan’s multiple-range test was applied to test differences among the treatments at *p* < 0.05 using Statistical Analysis Systems (SAS 9.13) software.

## Results

### Different Al Resistance in Cultivars of Pea

**FIGURE 1:**
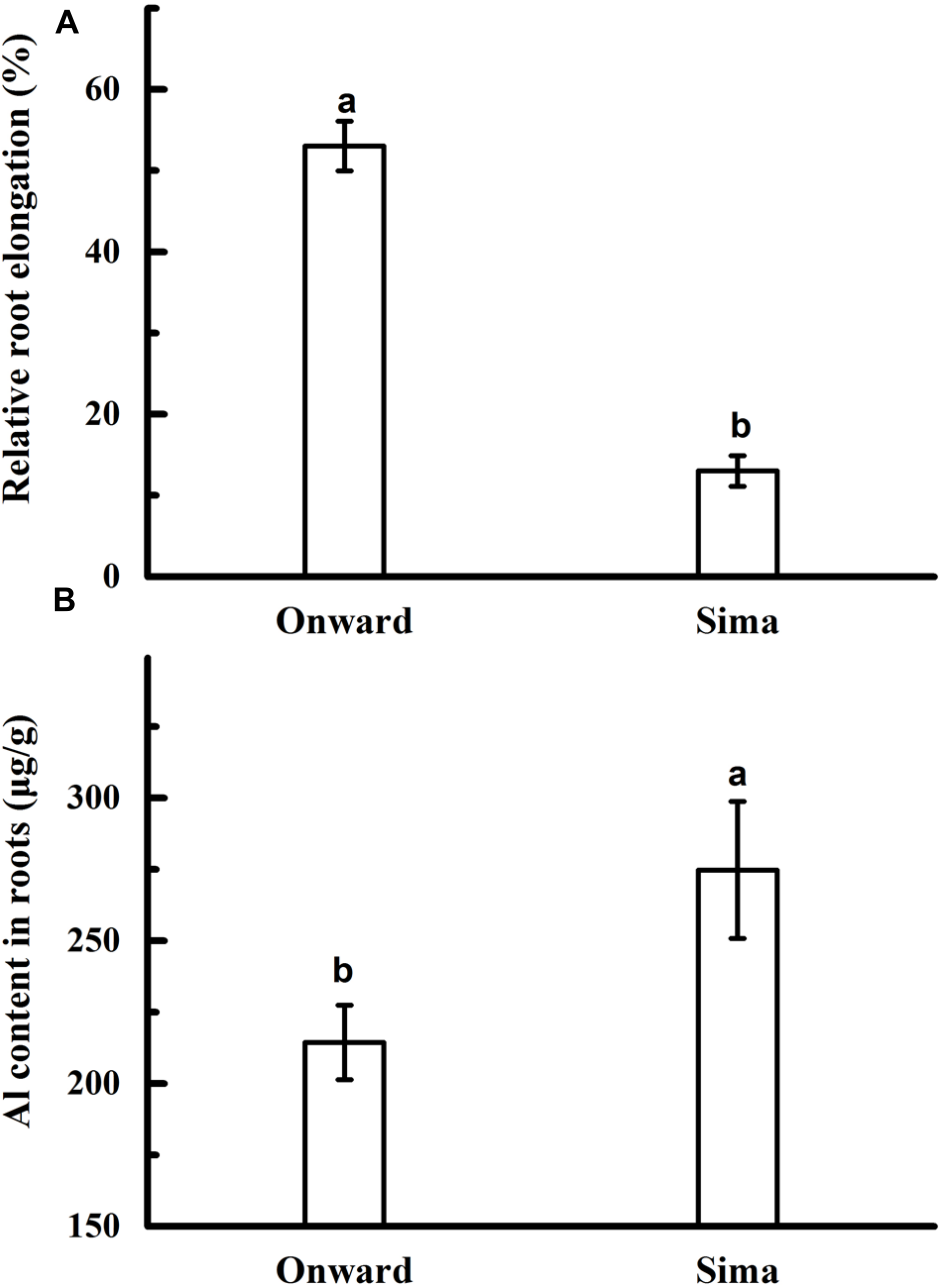
**Effect of Al application on root elongation (A) and Al content (B) in different cultivars of pea.** Six-day-old seedlings were exposed to 0 or 30 μM AlCl_3_ solution (pH 4.5, containing 0.5 mM CaCl_2_ and 25 μM H_3_BO_3_) for 24 h. The root length was measured before and after Al treatment. Then, 0–10.0 mm root tips were collected to determine Al content. Bars represent means ± SD, *n* = 4. Different letters indicate significant difference at *p* < 5%.

Root elongation and Al content was adopted to compare Al resistance in different cultivars of pea. Root elongation was inhibited by Al toxicity in both cv Onward and cv Sima, but RRE in cv Onward was higher than that in cv Sima (**Figure 1A**). Root elongation of cv Onward was inhibited by 47% after 24 h exposures to 30 μM AlCl_3_, whereas it was 87% for cv Sima (**Figure 1A**). Meanwhile there was significantly less Al accumulation in cv Onward comparing to cv Sima (**Figure 1B**). These results confirm that cv Onward is an Al-resistant cultivar while cv Sima is an Al-sensitive cultivar.

### Al Content in Roots

**FIGURE 2:**
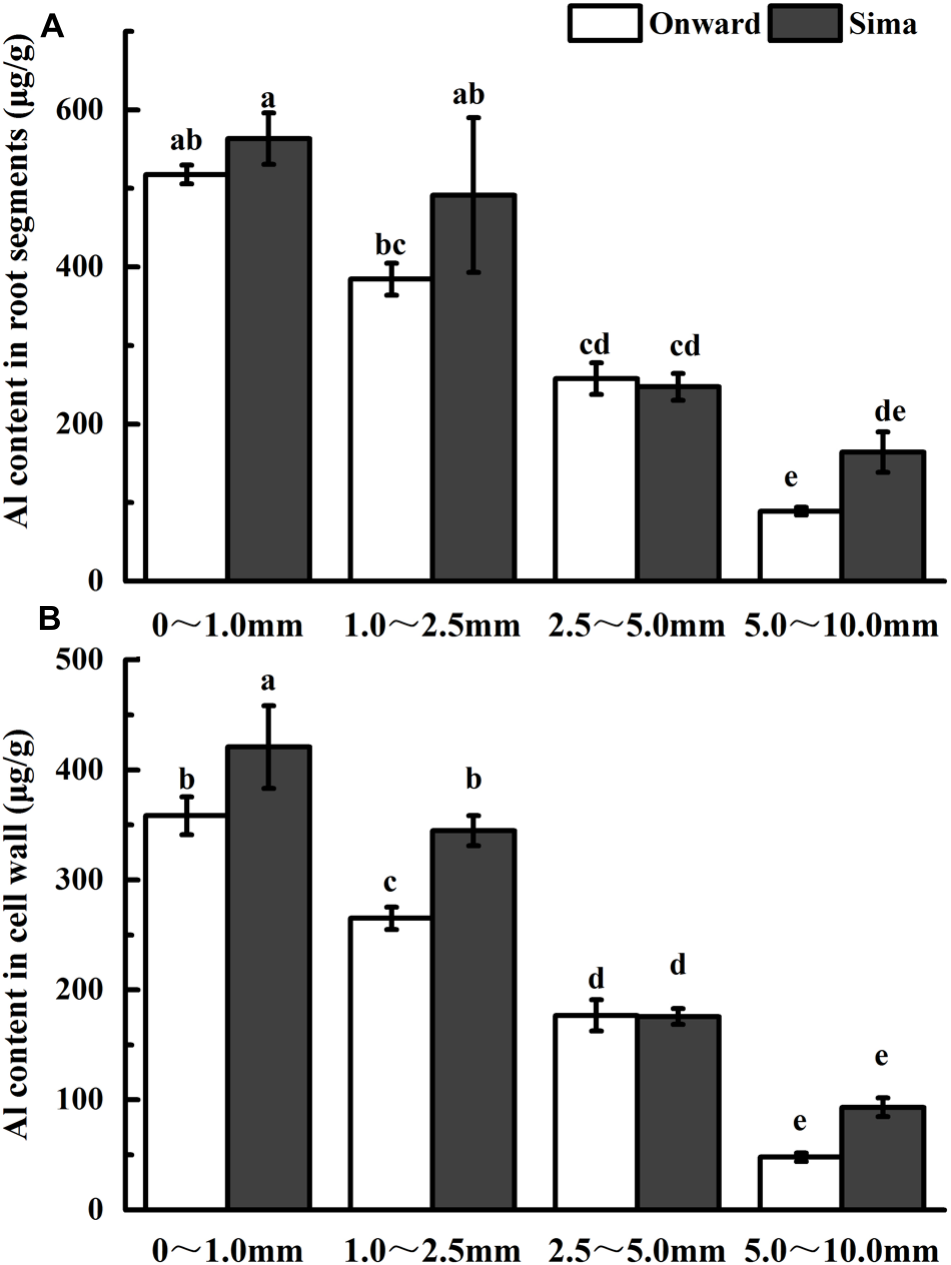
**Effect of Al application on Al content in root segments (A) or cell wall (B) in different cultivars of pea.** Six-day-old seedlings were exposed to 0 or 30 μM AlCl_3_ solution (pH 4.5, containing 0.5 mM CaCl_2_ and 25 μM H_3_BO_3_) for 24 h. Root segments were cut with sharp razor blades and Al content was determined by ICP-AES. Bars represent means ± SD, *n* = 4. Different letters indicate significant difference at *p* < 5%.

The content of Al in root segments or cell wall was measured in order to find the potential differences of Al accumulation in Al-resistant and Al-sensitive cultivars (**Figure 2**). Content of Al tended to decrease from root apex to root base both in root segment and cell wall. The Al accumulated in the cell wall accounted for about 70% of the total Al in the root, and there was a positive correlation between Al content in root and cell wall. Content of Al was higher in cv Sima than that in cv Onward at 0–1.0 mm and 1.0–2.5 mm root segment, and there was a significant differences in the cell wall. It indicates that Al accumulates mainly in root apex (0–1.0 mm and 1.0–2.5 mm segments), wherein more Al accumulates in cv Sima than in cv Onward in both the root cells and root cell wall.

### Morin Staining

**FIGURE 3:**
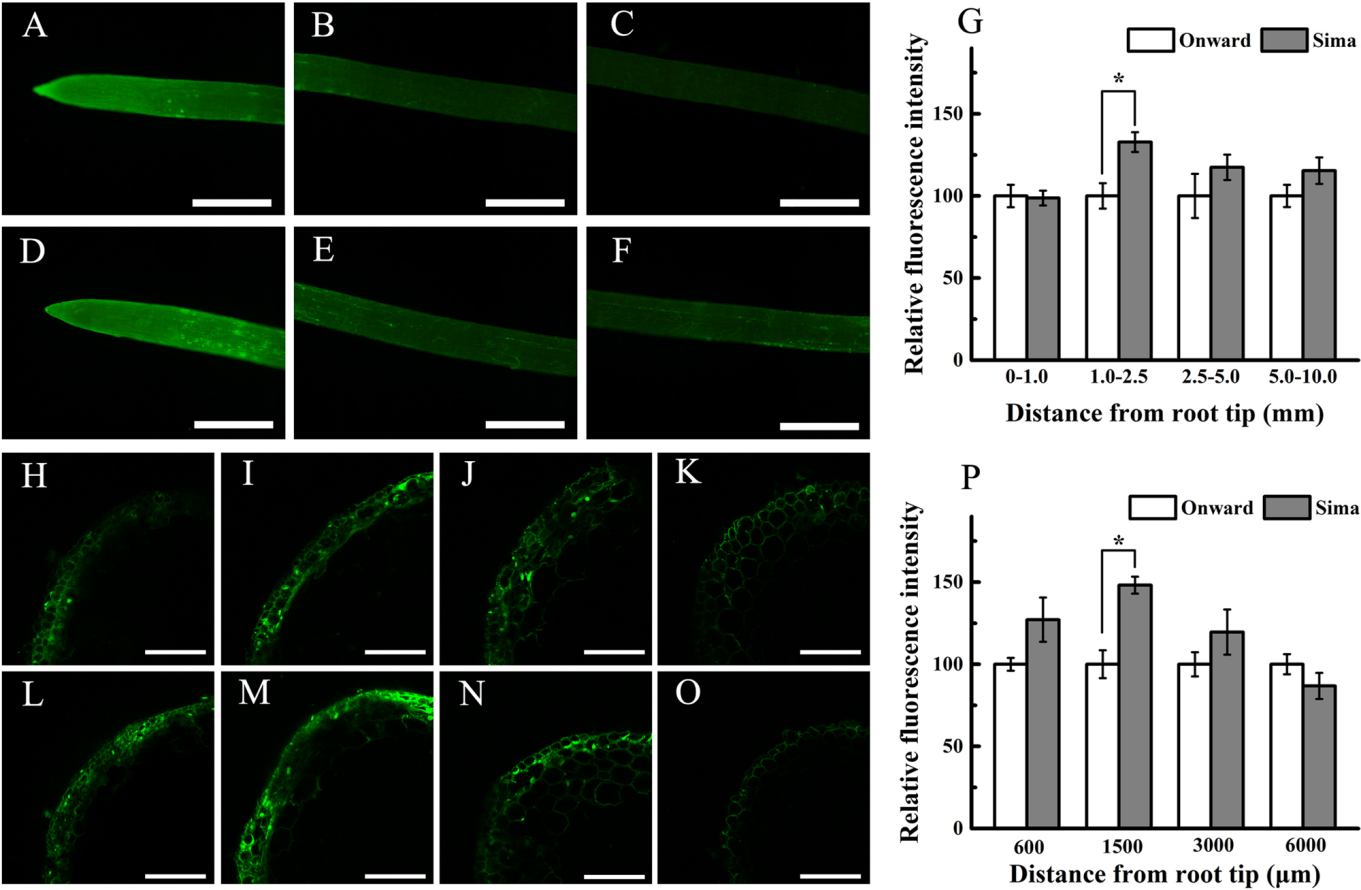
**The distribution of Al indicated by morin (green fluorescence) stain.** Roots were exposed to 30 μM AlCl_3_ (pH 4.5, containing 0.5 mM CaCl_2_ and 25 μM H_3_BO_3_) for 24 h. Roots of cv Onward **(A–C,H–K)** and cv Sima **(D–F,L–O)** was observed using an fluorescence microscope (whole root, **A–F**) and LSCM (root transverse section, **H–O**), respectively. Roots were transversely sectioned at 600 μm **(H,L)**, 1500 μm **(I,M)**, 3000 μm **(J,N)** and 6000 μm **(K,O)** from the apex for morin staining and fluorescence observation. Relative fluorescence intensity of cv Onward was used as reference 100% value **(G,P)**. Scale bars = 1 mm **(A–F)** or 100 μm **(H–O)**. Asterisks above columns indicate statistically significant differences at the same segment between cv Onward and cv Sima (*p* < 5%).

Morin is an appropriate dye to study qualitatively the radial Al distribution along the root tip axis (Klug et al., 2011). Some recent study indicates that morin can detect Al in the cytosol but not cell wall-bound Al or vacuole-compartmentalized Al (Eticha et al., 2005b; Huang et al., 2012), and strong Al-dependent green fluorescence represents Al present in the cytosol and nucleus. To gain further evidence for possible distribution of Al, we localized Al with morin staining. After exposure to 30 μM Al for 24 h, roots showed stronger fluorescence at 0–3 mm root tips (**Figures 3A,D**) than the other root segments (**Figures 3B,C,E,F**) in both cv Onward and cv Sima. There was brighter fluorescence at 1.0–2.5 mm root of cv Sima than that of cv Onward (**Figures 3A,D**). Through the semi-quantitative calculation of fluorescence intensity, the data showed that the fluorescence intensity of cv Sima was significantly higher than that of cv Onward at 1.0–2.5 mm root segments (**Figure 3G**). Then morin stain was applied in root transverse section at 600, 1500, 3000, and 6000 μm from the apex. The Al-sensitive cv Sima displayed stronger Al-dependent green fluorescence than the Al-resistant cv Onward at 600, 1500, and 3000 μm (**Figures 3H–P**). There was significant difference in the fluorescent intensity counted at 1500 μm of cv Sima than that of cv Onward. In the meantime we found the green fluorescence mainly appeared in the epidermis and outer cortical cell layers in both cultivars. However, the green fluorescence of morin could be seen in more cell layers to the root axis at 1500 and 3000 μm of cv Sima than that of cv Onward. These results show that cv Sima accumulates more Al in the cytosol at the transition zone than cv Onward.

### Effect of Al Treatment on Pectin

**FIGURE 4:**
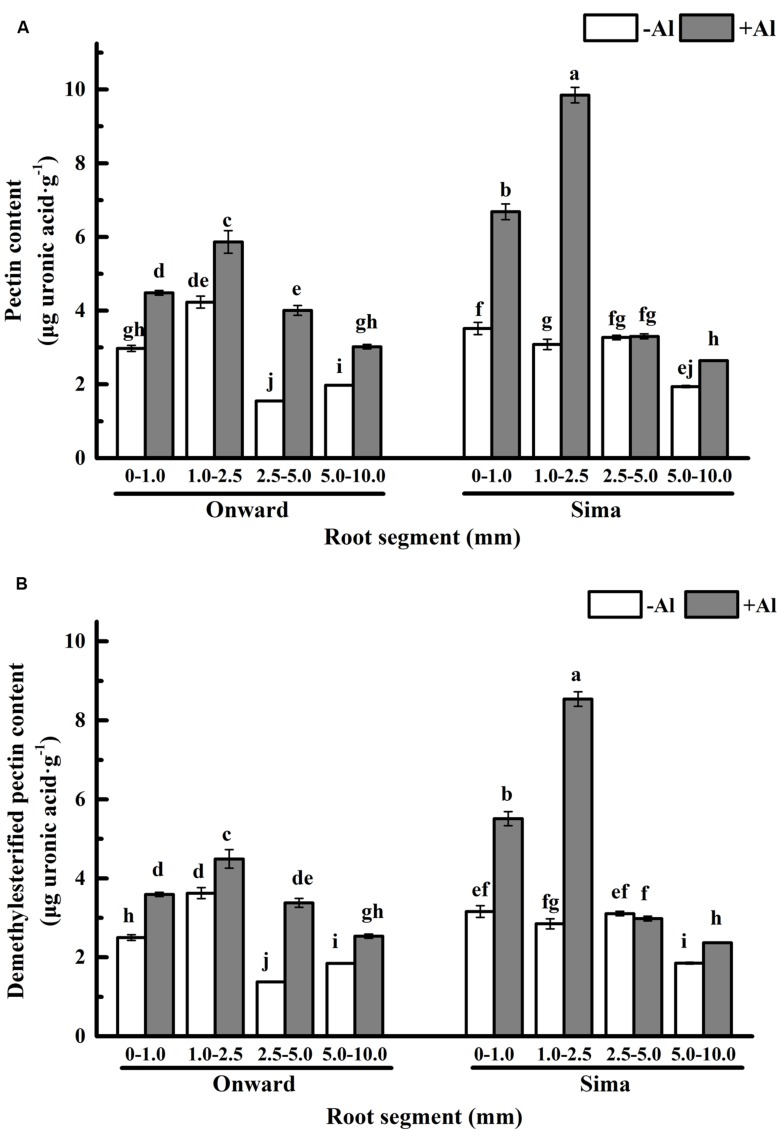
**Pectin content in different root segments of different cultivars of pea (*Pisum sativum*).** Six-day-old seedlings were exposed to 0 or 30 μM AlCl_3_ solution (pH 4.5, containing 0.5 mM CaCl_2_ and 25 μM H_3_BO_3_) for 24 h. **(A)** The pectin content of cell wall. **(B)** The demethylesterified pectin content. The demethylesterified pectin content was calculated by the formula of content of demethylesterified pectin = pectin content × (1-DM/100). Bars represent means ± SD, *n* = 4. Different letters indicate significant difference at *p* < 5%.

Pectin is the major component of cell wall that binds Al (Horst, 1995; Chang et al., 1999), which may define the Al sensitivity of cultivars (Yang et al., 2011b). In our results, the pectin distribution trend in roots was almost same in both cv Sima and cv Onward (**Figure 4A**). The pectin content was increased in mostly root segments after Al treatment, and the increase was mainly in 0–1.0 mm and 1.0–2.5 mm segments, it was 50% (from 3.0 μg/g to 4.5 μg/g) and 39 % (from 4.2 μg/g to 5.9 μg/g) in cv Onward, 90% (from 3.5 μg/g to 6.7 μg/g) and 219% (from 3.1 μg/g to 9.8 μg/g) in cv Sima, respectively. The increase of pectin in cv Sima was extraordinarily higher than that in cv Onward, especially in 1.0–2.5 mm segments. It indicates that the pectin metabolism is more responsive to Al toxicity in cv Sima. Therefore, Al exposure stimulates the increase of pectin in both cultivars and it is more prominent in Al-sensitive cultivar, especially in the transition zone (1.0–2.5 mm).

The primary binding sites of Al^3+^ in pectic matrix relies on its carboxylic groups, which have large numbers negatively charged and a particularly high affinity for Al^3+^ (Blamey et al., 1990; Chang et al., 1999). The number of binding sites are essentially determined by pectin content and degree of methyl-esterification. The content of demethylesterified pectin was computed by the formula that content of demethylesterified pectin = pectin content × (1–DM/100), DM was expressed in percentage (%) and was calculated as the moles of methanol per mol of galacturonic acid. The overall trend of demethylesterified pectin content remained the same as pectin content in both cultivars (**Figure 4B**). It indicates that Al induces significantly more demethylesterified pectin in root transition zone (and meristem and root caps) of Al sensitive cv Sima than that in Al resistant cv Onward.

### Effect of Al Treatment on the Degree of Pectin Methyl-esterification

**FIGURE 5:**
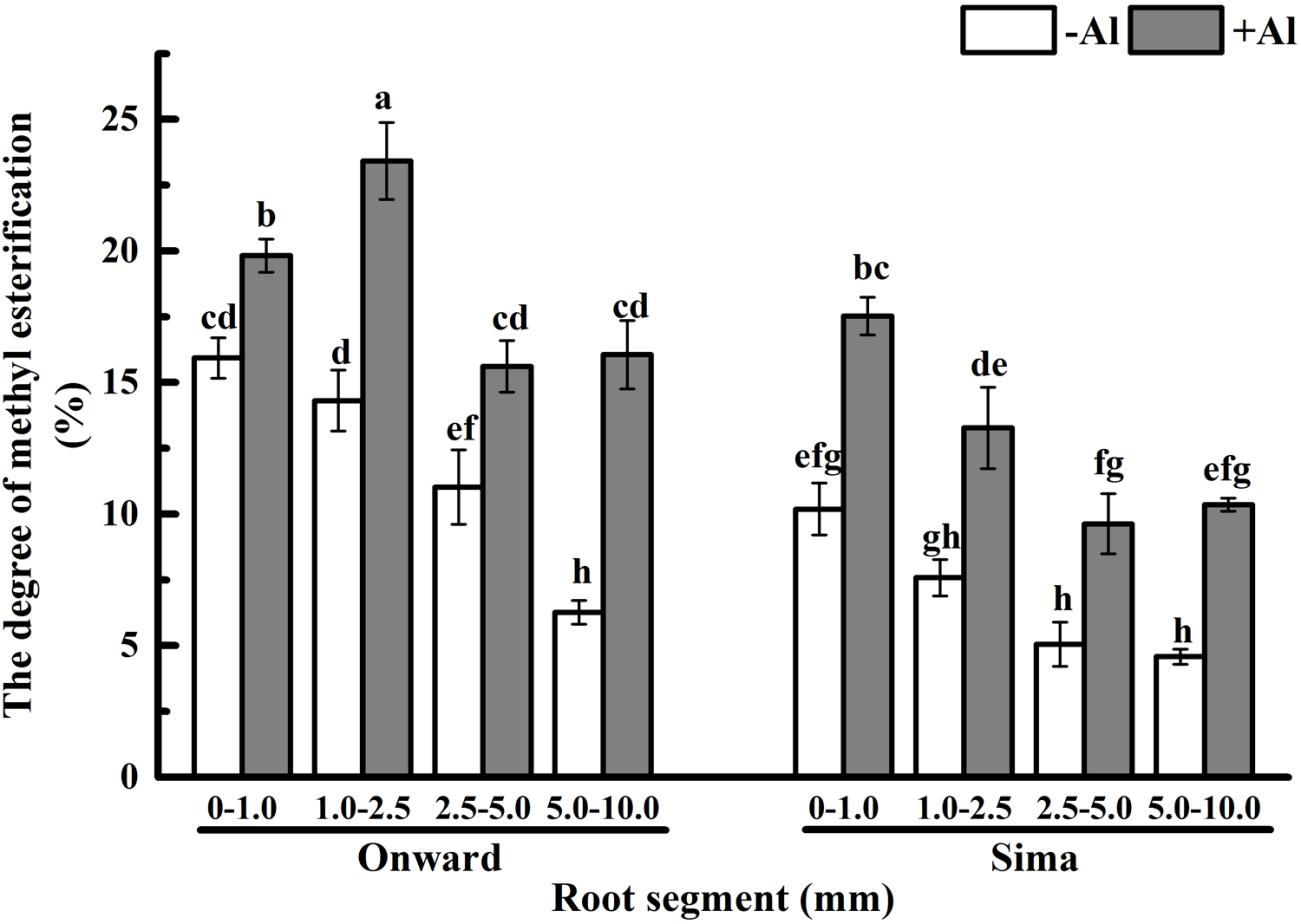
**Effect of Al stress on the degree of pectin methyl-esterification in root segments of different cultivars of pea (*Pisum sativum*).** Six-day-old seedlings were exposed to 0 or 30 μM AlCl_3_ solution (pH 4.5, containing 0.5 mM CaCl_2_ and 25 μM H_3_BO_3_) for 24 h. Different root segments were collected to determine the degree of pectin methyl-esterification based on the concentration of uronic acid and methanol. The degree of methyl-esterification (DM, %) was calculated as moles of methanol per mol of galacturonic acid. Bars represent means ± SD, *n* = 4. Different letters indicate significant difference at *p* < 5%.

Many evidences indicate that not only pectin content contributes to Al accumulation in plants but also the degree of pectin methyl-esterification (DM) which determines the ratios of negatively charged carboxylic groups to bind Al (Eticha et al., 2005a; Rangel et al., 2009; Yang et al., 2011b). Degree of pectin methyl-esterification in root apex ranged from 25 to 5% in different segments and tended to decrease from root apex to root base (**Figure 5**), it was higher in Al-resistant cultivar than that in Al-sensitive cultivar. After Al treatment for 24 h, the degree of pectin methyl-esterification increased significantly in both cultivars, while it was still higher in cv Onward than cv Sima. The degree of pectin methyl-esterification in 1.0–2.5 mm roots was highest in cv Onward, which was 1.76 folds to that of cv Sima. The degree of pectin methyl-esterification was highest in 0–1.0 mm root segments of cv Sima instead of 1.0–2.5 mm root segments in cv Onward. These results indicate that Al promote the degree of pectin methyl-esterification increase in both Al-resistant cv Onward and cv Al-sensitive cv Sima.

### Effect of Al Treatment on PME

**FIGURE 6:**
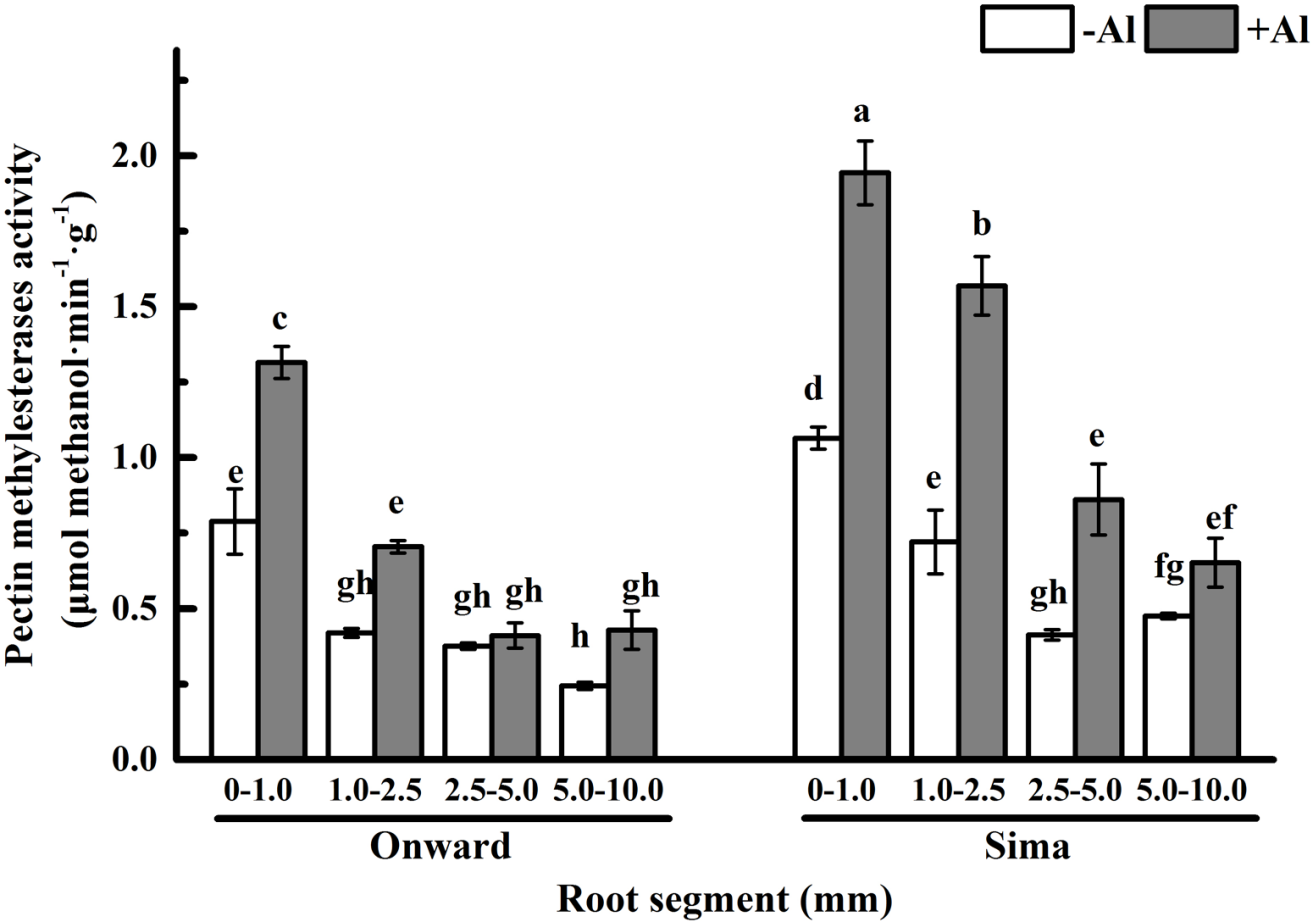
**Effect of Al stress on PME activity in root segments of different cultivars of pea (*Pisum sativum*).** Six-day-old seedlings were exposed to 0 or 30 μM AlCl_3_ solution (pH 4.5, containing 0.5 mM CaCl_2_ and 25 μM H_3_BO_3_) for 24 h. Different root segments were collected to determine PME activity as described in Section “Materials and Methods.” Bars represent means ± SD, *n* = 4. Different letters indicate significant difference at *p* < 5%.

Pectin is mainly demethylesterificated by PME and the degree of pectin methyl-esterification are mainly determined by PME (Micheli, 2001). The activity of PME in 0–1.0 mm root was the highest and decreased basipetally from the root apex in both cultivars (**Figure 6**), which is consistent with the process of pectin maturation along root axis. The activity of PME was significantly higher in cv Sima than in cv Onward no matter with or without Al. However, Al treatment induced a significant increase of PME activity and the increase was more prominent in Al-sensitive cultivar, especially at the 1.0–2.5 mm root segments. The activity of PME in 1.0–2.5 mm root segments of Al-sensitive cv Sima was 2.2 folds to that of Al-resistant cv Onward.

## Discussion

The initial symptoms of Al toxicity in plants are the rapid inhibition of root elongation. The extent of root growth inhibition has been used extensively as a criterion for Al toxicity and Al resistance (Foy, 1988). In the present study, Al-induced inhibition of root elongation and the accumulation of Al was adopted to distinguish cultivars of pea into Al-resistant cv Onward and Al-sensitive cv Sima (**Figure 1**). Cultivar Onward displays higher RRE due to lower Al accumulation in comparison with cv Sima (**Figure 2**).

Morin staining interestingly shows that root transition zone (1.0–2.5 mm roots) displays stronger Al-dependent green fluorescence than the other segments, which is stronger in cv Sima than in cv Onward (**Figure 3**). The transverse distribution of Al-dependent green fluorescence in transverse root section also indicates that more Al enters into cytosol of root transition zone than the other zones, especially for cv Sima. Aluminum in the cytosol is more toxic than in the cell wall (Delhaize and Ryan, 1995). It may contribute to the sensitivity of the root transition zone to Al toxicity and Al resistance of the cultivars of pea (*Pisum sativum* L.).

Our work disclosed how the transition zone determined the sensitivity/resistance of the cultivars focusing on the properties of cell wall which was the main target of Al binding.

### The Pectin of Transition Zone is most Prominent to Al-induced Increase

The analysis of Al content indicates that Al accumulates predominately in 0–1.0 mm and 1.0–2.5 mm root segments of pea (**Figure 2A**), and cell wall is the major target of Al accumulation (**Figure 2B**). Al accumulates primarily and predominantly in the root apoplast because the pectin matrix has negative charges to bind Al (Schmohl and Horst, 2000; Horst et al., 2010; Yang et al., 2011b). The Al binding to the cell wall pectin-matrix modulates Al sensitivity (Schmohl and Horst, 2000). The results show that, at the absence of Al, there is no essential difference in pectin content between cv Sima and cv Onward. After 24 h Al treatment, pectin content increases significantly in the two cultivars, however, the increase of pectin content is more prominent in the Al-sensitive cv Sima than in the Al-resistant cv Onward. As a result, after Al treatment, the content of pectin is significantly higher in cv Sima than in cv Onward, especially the transition zone. This is consistent with the result in maize (Eticha et al., 2005a) that cell wall pectin contributes to genotypic differences in Al resistance.

Therefore, responses of pectin to Al toxicity distinguish the 1.0–2.5 mm root from the other segments to be most sensitive to Al toxicity, which also distinguish Al-sensitive cv Sima from the Al-resistant cv Onward (**Figures 4** and **5**). Al-induced 3.2 folds and 1.4 folds increase of pectin in 1.0–2.5 mm roots of cv Sima and cv Onward respectively. Therefore, pectin content in 1.0–2.5 mm root segments of cv Sima is significantly higher than that of cv Onward after Al exposure. The similar trend of demethylesterified pectin content was found in root segments.

Sivaguru and Horst (1998) demonstrate that the distal part of the transition zone is the most Al-sensitive root zone in maize and the transition zone could sense the presence of Al and further regulate root elongation. The transition zone, as the most sensitive zone, has the highest content of pectin and demethylesterified pectin (**Figure 4B**). The large number of negative charges on the demethylesterified pectin is generally considered to be conducive to the accumulation of Al^3+^ (Schmohl and Horst, 2000). However, 1.0–2.5 mm segments have lower Al content compared with 0–1.0 mm root segments (**Figure 2**). In the 2.5–5.0 mm segments, the green fluorescence of cv Sima was higher than that of cv Onward, but Al content in roots or cell wall show no differences. This may be the result of the redistribution of aluminum. We speculate that there may be other factors affecting the distribution of Al in the apical cell wall, e.g., the structure of pectin and the pH of root surface, which require further study.

### Pectin and PME in Root Transition Zone Cooperate to Determine Al Resistance in Cultivars of Pea

In recent years, some evidences suggest that Al toxicity induces cellular damage via cell wall-plasma membrane-cytoskeleton continuum and thus root cell wall plays an important role in Al resistance (Horst et al., 1999, 2010; Sivaguru et al., 1999). The Al-binding capacity of cell wall pectin depends on both pectin content and the degree of pectin methyl-esterification (Eticha et al., 2005a,b; Kyomugasho et al., 2015). In order to expound the effect of pectin properties on Al sensitivity, the degree of pectin methyl-esterification and PME activity in different root zones were compared between Al-sensitive and Al-resistant cultivars. The results show that Al toxicity not only promotes the increase of pectin, but also increase the degree of pectin methyl-esterification and the PME activity (**Figures 5** and **6**). The increase of degree of pectin methyl-esterification and the PME activity may be attributed to the promotion of Al on synthesis of pectin. The newly born pectin is highly esterified, therefore elevates the degree of pectin methyl-esterification. The increase of pectin synthesis also prompts the increase of the PME activity. Since PME activity of cv Sima is always higher than cv Onward, the degree of pectin methyl-esterification is higher in cv Onward than in cv Sima, especially in 1.0–2.5 mm root segments. We have reasons to believe that Al resistance in cv Onward is related to the lower content of pectin and lower activity of PME in root transition zone, especially in the presence of Al.

## Conclusion

Our results clearly demonstrate that Al promotes the pectin synthesis in pea root tip, and accompanied with the increase of PME activity and degree of esterification. The PME activity of Al-sensitive cv Sima is higher than that of Al-resistant cv Onward, especially for the transition zone. In the transition zone of Al-sensitive cultivar, which is the most sensitive zone to Al, the most prominent to Al-induced pectin increase and the higher PME activity results in higher content of demethylesterified pectin and higher Al accumulation in cell wall and cytosol. Therefore we have reasons to believe that the transition zone contributes, at least in part, to the differential Al resistance among cultivars. Further studies are required to reveal the details of Al redistribution in root cells of root transition zone of Al-resistant cultivars and Al-sensitive cultivars.

## Author Contributions

LW and MY designed experiments; XL, YL, and MQ carried out experiments; XL and HX analyzed experimental results. XL wrote the manuscript. YF, JL, and MY modified the manuscript.

## Conflict of Interest Statement

The authors declare that the research was conducted in the absence of any commercial or financial relationships that could be construed as a potential conflict of interest.
