# Supplementary material for: Cell Wall Pectin and its Methyl-esterification in Transition Zone Determine Al Resistance in Cultivars of Pea (Pisum sativum)
Source: Front Plant Sci. 2016 Feb 1;7:39. doi: 10.3389/fpls.2016.00039 (PMC4734104; doi:10.3389/fpls.2016.00039)
Supplement: Supplementary file 1 [file Table_1.DOCX]

**Supplemental data**

Table S1. Growth zones in the pea root apex.

| Length (μm) | Root Zone | | |
| --- | --- | --- | --- |
|  | Root Cap | Meristem Zone | Transition Zone |
| End Scope | 360-521 | 938-1458 | 1875-3135 |
| Average | 369±6 | 1188±20 | 2242±41 |
| Sample Number | n=170 | n=134 | n=109 |

The 0-10 mm roots of six-day-old pea seedlings were collected and different zone was measured.
